# Supplementary material for: Mts1 (S100A4) and Its Peptide Demonstrate Cytotoxic Activity in Complex with Tag7 (PGLYRP1) Peptide
Source: Int J Mol Sci. 2024 Jun 16;25(12):6633. doi: 10.3390/ijms25126633 (PMC11203719; doi:10.3390/ijms25126633)
Supplement: Supplementary file 1 [file ijms-25-06633-s001.zip › ijms-3036268-supplementary.pdf]

Supplementary Materials
"Mts1(S100A4) and its peptide acquire anticancer cytotoxic activity in complex with Tag7(PGLYRP1) peptide."

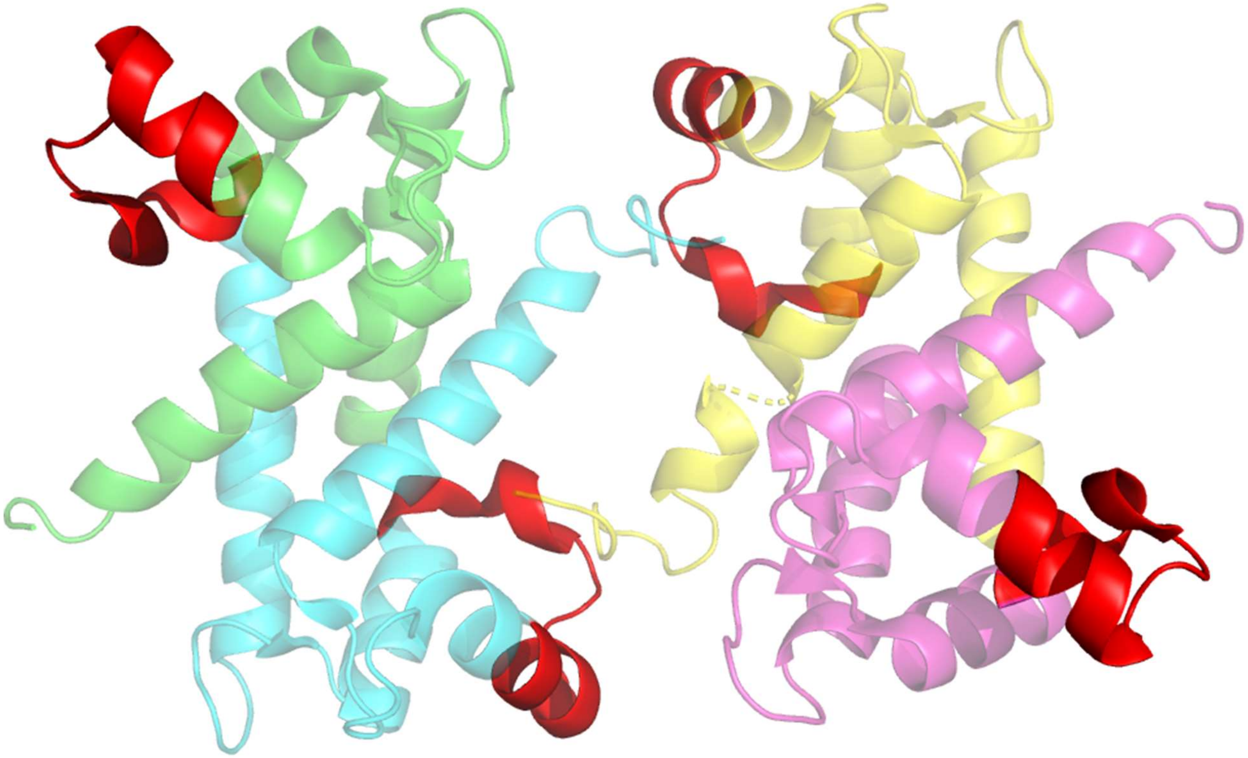

Figure S1. *The Mts1 protein*. Subunit A is *green*, subunit B is *blue*, subunit C is *magenta*, subunit D is *yellow*. The four identical secondary structures (see the main text) formed by the portion of Mts corresponding to the M7 peptide are colored in *intense red*. The remaining portion of the protein is semi transparent.

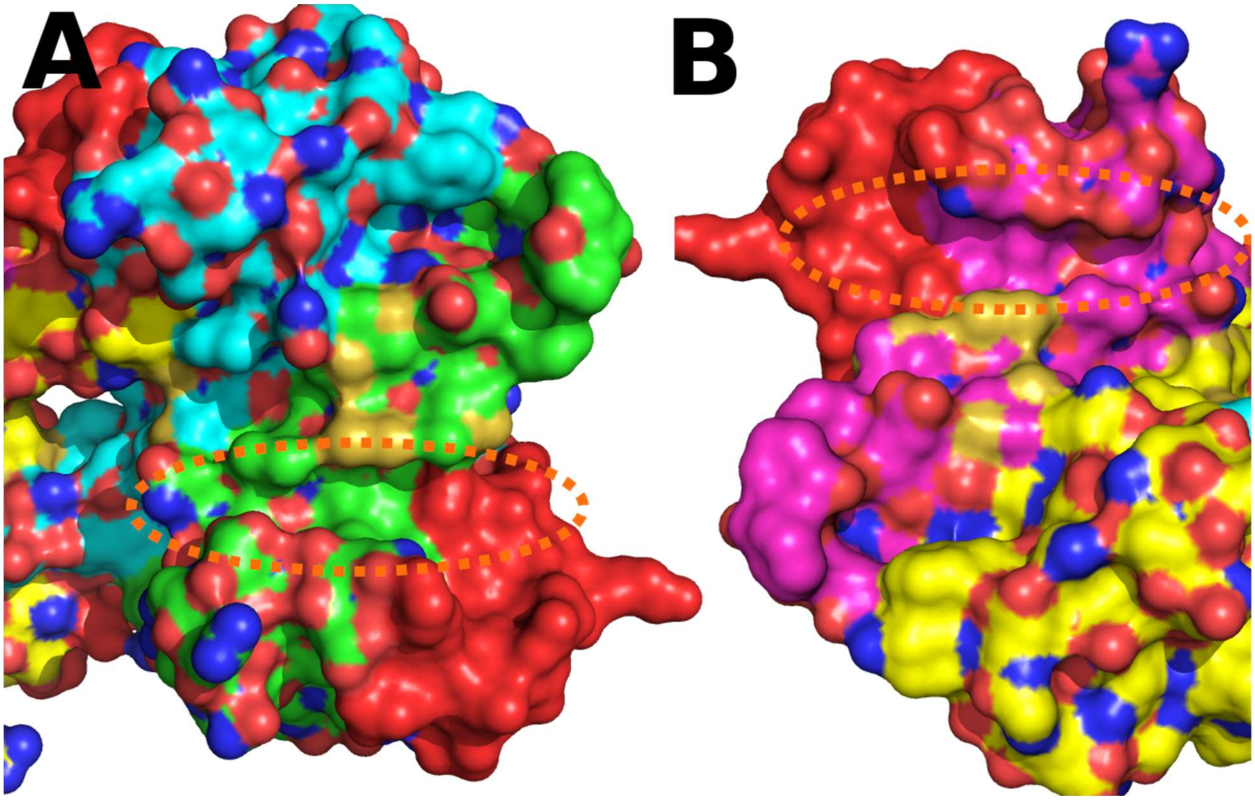

Figure S2. *Possible binding sites for 17.1 peptide on the surface of Mts1*. Grooves that may possibly form the binding sites for the 17.1 are encircled with *orange*. Color scheme is identical to the one from Figure S1.

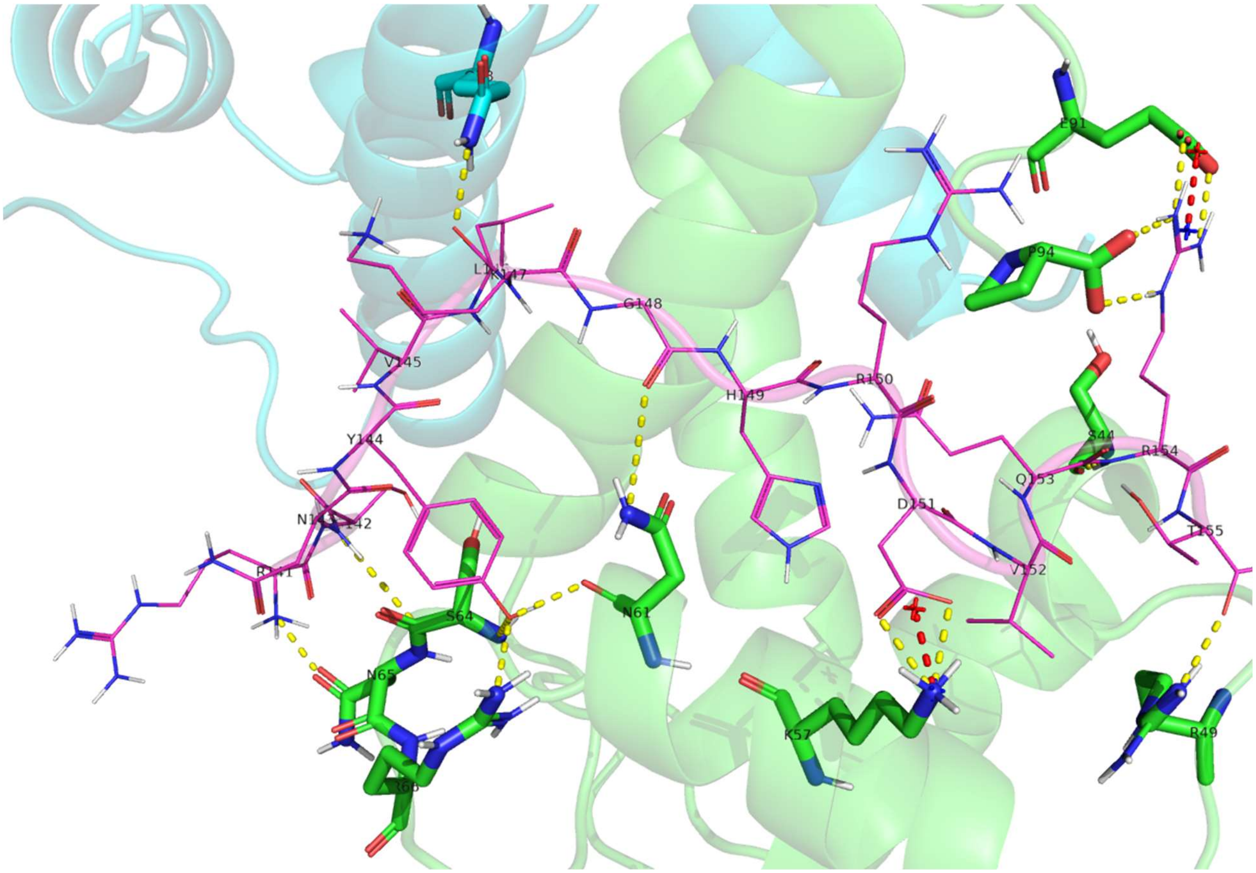

Figure S3. *Interactions between the 17.1. peptide and the Mts1 protein*. Only hydrogen bonds (*yellow dashed lines*) and salt bridges (*red dashed lines*) are shown. Peptide`s residues are shown as *thin sticks*, backbone is *pale cartoon*. MTS1 is represnted by *pale cartoon* with interacting residues as *bold sticks,* subunit A is *green*, subinit B is *blue*.

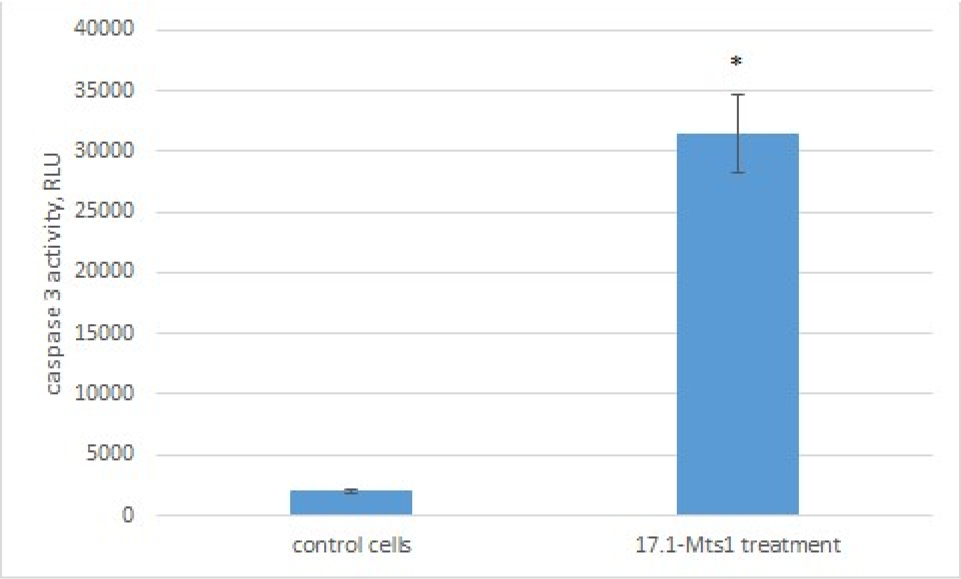

Figure S4. Caspase 3 activity assay. The Caspase 3 activity was determined using the Caspase 3 Assay kit, Fluorimetric (Sigma-Aldrich, St. Louis, MO, USA). For this, 10^7 cells were treated with 17.1-Mts1 for 3 h at 37 °C. After this period, 5 µL of the reagent AMC was added to each well and, after homogenization, samples were incubated for 1 h at room temperature. Then, caspase 3 activity was determined by luminescence (CLARIOstar Plus, BMG LABTECH GmbH, Ortenberg, Germany).

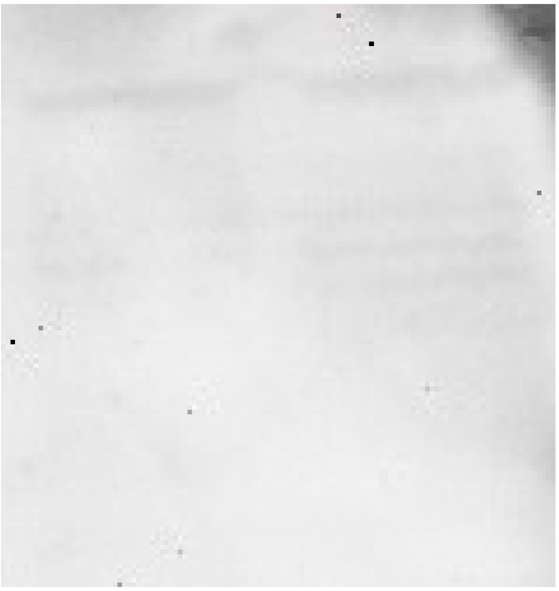

Figure S5. (b) Western blot with antibodies to caspase 9 from cell lysates 1 hour after 17.1-Mts1 addition (left) and control cells (right)

Table S1. Interactions detected between the 17.1. peptide and the Mts1 protein represented by the most populated cluster from MD trajectory. *HBDonor* and *HBAcceptor* stands for "hydrogen bond donor" and "hydrogen bond acceptor" respectively. *Hydrophobic*, *anionic* and *cationic* are self-explaining. Interactions between M7 portion of Mts1 and 17.1A of 17.1 are in *green* font.

17.1 residue	Mts1 residue	Interaction type
ARG141	ASN65	HBDonor
ASN143	SER64	HBDonor
TYR144	ASN61	HBDonor
	SER64	HBDonor
	ARG66	HBAcceptor
VAL145	VAL77	Hydrophobic
	VAL77	Hydrophobic
LEU146	VAL77	Hydrophobic
	CYS81	Hydrophobic
	MET84	Hydrophobic
	GLN73	Hydrophobic
		HBAcceptor
	CYS76	Hydrophobic
	VAL77	Hydrophobic
GLY148	ASN61	HBAcceptor
HIS149	LEU58	Hydrophobic
	ASN61	Hydrophobic
ARG150	PRO94	Hydrophobic
ASP151	LYS57	Hydrophobic
	LYS57	HBAcceptor
	LYS57	Anionic
VAL152	ALA54	Hydrophobic

	LYS57	Hydrophobic
	LEU58	Hydrophobic
GLN153	PHE45	Hydrophobic
	LEU58	Hydrophobic
	PRO94	Hydrophobic
ARG154	SER44	HBDonor
	GLU91	HBDonor
	GLU91	Cationic
	PHE93	Hydrophobic
	PHE93	HBDonor
	PRO94	HB Donor
THR155	ARG49	HBAcceptor

Table S2. Interactions detected between the 17.1. peptide and the TNFR protein represented by the cluster 1 from MD trajectory. *HBDonor* and *HBAcceptor* stands for "hydrogen bond donor" and "hydrogen bond acceptor" respectively. *Hydrophobic*, *anionic* and *cationic* are self-explaining. Residues of 17.1 that interact with TNFR in representative conformations of every cluster are in *orange* font.

17.1 residue	TNFR residue	Interaction type
ARG141	GLU56	HBDonor
		Cationic
ASN143	GLU56	HBDonor
	SER57	HBDonor
	SER59	HBDonor
	HIS69	Hydrophobic
TYR144	GLU56	Hydrophobic
	SER57	HBAcceptor
		HBDonor

HIS149	LEU71	Hydrophobic
ARG150	PHE60	Hydrophobic
	SER74	HBDonor
	ASP93	HBDonor
		Cationic
	ASN110	HBDonor
ASP151	ARG77	HBAcceptor
		Anionic
	ASN110	HBAcceptor
VAL152	LYS75	HBAcceptor
	ARG77	Hydrophobic
GLN153	SER72	HBAcceptor
ARG154	LYS75	Hydrophobic
		HBAcceptor
	CYS76	HBDonor
	GLU79	HBDonor
		Cationic
THR155	LYS75	HBAcceptor

Table S3. Interactions detected between the 17.1. peptide and the TNFR protein represented by the cluster 2 from MD trajectory. *HBDonor* and *HBAcceptor* stands for "hydrogen bond donor" and "hydrogen bond acceptor" respectively. *Hydrophobic*, *anionic* and *cationic* are self-explaining. Residues of 17.1 that interact with TNFR in representative conformations of every cluster are in *orange* font.

17.1 residue	TNFR residue	Interaction type
ASN143	GLU56	Hydrophobic
		HBDonor
	SER59	HBDonor

TYR144	GLU56	Hydrophobic
		HBDonor
HIS149	SER72	HBDonor
ARG150	LYS75	HBAcceptor
	ASN110	HBDonor
ASP151	LYS75	HBAcceptor
	ARG77	HBAcceptor
		Anionic
	ASN110	HBAcceptor
VAL152	LYS75	HBAcceptor
	ARG77	Hydrophobic
GLN153	LYS75	HBAcceptor
	GLU79	HBDonor
ARG154	CYS73	HBDonor
	SER74	HBDonor
	LYS75	Hydrophobic
THR155	LYS78	HBAcceptor
	GLU79	HBDonor

Table S4. Interactions detected between the 17.1. peptide and the TNFR protein represented by the cluster 3 from MD trajectory. *HBDonor* and *HBAcceptor* stands for "hydrogen bond donor" and "hydrogen bond acceptor" respectively. *Hydrophobic*, *anionic* and *cationic* are self-explaining. Residues of 17.1 that interact with TNFR in representative conformations of every cluster are in *orange* font.

17.1 residue	TNFR residue	Interaction type
ARG141	GLU56	HBDonor
		Cationic
ASN143		HBDonor

	SER59	HBDonor
	HIS69	Hydrophobic
	SER72	HBAcceptor
TYR144	SER57	HBDonor
	SER72	HBDonor
HIS149	PHE60	Hydrophobic
	LEU71	Hydrophobic
ARG150	ASP93	HBDonor
		Cationic
ASP151	SER74	HBAcceptor
	ARG77	HBAcceptor
		Anionic
VAL152	LYS75	HBAcceptor
	ARG77	Hydrophobic
GLN153	SER72	HBAcceptor
ARG154	LYS75	Hydrophobic
		HBAcceptor
	CYS76	HBDonor
	GLU79	HBDonor
		Cationic
THR155	LYS75	HBAcceptor

Table S5. Interactions detected between the 17.1. peptide and the TNFR protein represented by the cluster 4 from MD trajectory. *HBDonor* and *HBAcceptor* stands for "hydrogen bond donor" and "hydrogen bond acceptor" respectively. *Hydrophobic*, *anionic* and *cationic* are self-explaining. Residues of 17.1 that interact with TNFR in representative conformations of every cluster are in *orange* font.

17.1 residue	TNFR residue	Interaction type
ARG141	GLU56	HBDonor
		Cationic
TYR144	GLU56	Hydrophobic
		HBDonor
LYS147	SER72	HBAcceptor
ARG150		HBDonor
	CYS73	HBDonor
	SER74	HBDonor
	ASP93	HBDonor
		Cationic
ASP151	ARG77	HBAcceptor
		Anionic
VAL152	LYS75	Hydrophobic
		HBAcceptor
	ARG77	Hydrophobic
GLN153	SER72	HBDonor
	CYS73	HBDonor
ARG154	LYS75	Hydrophobic
		HBAcceptor
	GLU79	HBDonor
		Cationic
THR155	LYS75	HBAcceptor

Supplemental MALDI Peak Table.

Peptide,-10lgP,Mass,Length,ppm,m/z,RT,Area Sample 3,Fraction,Scan,Source File,#Feature,#Feature Sample 3,Accession,PTM,AScore,Found By
HSQAVEELAEQLEQTK,55.49,1838.9010,16,1.4,613.9752,71.25,2.3923E5,5,23979,Yashin_004_20230531_ZR_DY-3.raw,1,1,A0A024R1N1|A0A024R1N1_HUMAN:sp|P35579|MYH9_HUMAN:A0A8I5KWT8|A0A8I5KWT8_HUMAN:A0A0U4BW16|A0A0U4BW16_HUMAN:Q86XU5|Q86XU5_HUMAN,,,PEAKS DB
LQQELDDLLVDLD-HQR,53.46,1948.9854,16,2.3,650.6705,73.29,3.6998E5,5,24956,Yashin_004_20230531_ZR_DY-3.raw,2,2,A0A024R1N1|A0A024R1N1_HUMAN:sp|P35579|MYH9_HUMAN:A0A8I5KWT8|A0A8I5KWT8_HUMAN:A0A0U4BW16|A0A0U4BW16_HUMAN,,,PEAKS DB
VSHLLGINVTDFTR,52.93,1570.8467,14,2.0,524.6239,72.55,7.9479E4,5,24595,Yashin_004_20230531_ZR_DY-3.raw,1,1,A0A024R1N1|A0A024R1N1_HUMAN:sp|P35579|MYH9_HUMAN:A0A8I5KWT8|A0A8I5KWT8_HUMAN:Q86XU5|Q86XU5_HUMAN:sp|P35579-2|MYH9_HUMAN:B4E3S1|B4E3S1_HUMAN,,,PEAKS DB
SGGGGGGGLGSGG-SIR,51.69,1231.5905,16,1.4,616.8033,37.86,3.1795E6,5,10670,Yashin_004_20230531_ZR_DY-3.raw,1,1,sp|P35527|K1C9_HUMAN,,,PEAKS DB
HSQAVEELAEQLEQTKR,51.41,1995.0021,17,1.8,666.0092,69.85,2.7816E5,5,23327,Yashin_004_20230531_ZR_DY-3.raw,1,1,A0A024R1N1|A0A024R1N1_HUMAN:sp|P35579|MYH9_HUMAN:A0A8I5KWT8|A0A8I5KWT8_HUMAN:A0A0U4BW16|A0A0U4BW16_HUMAN:Q86XU5|Q86XU5_HUMAN,,,PEAKS DB
ELLTRELPSFLGK,50.70,1501.8503,13,2.3,751.9342,72.73,9.1911E5,5,24671,Yashin_004_20230531_ZR_DY-3.raw,2,2,sp|P26447|S10A4_HUMAN,,,PEAKS DB
ELPSFLGKRTDE-AAFQK,49.70,1936.0054,17,4.4,485.0107,68.33,1.0587E8,5,22782,Yashin_004_20230531_ZR_DY-3.raw,3,3,sp|P26447|S10A4_HUMAN,,,PEAKS DB
GNFNYIEFTR,49.64,1259.5935,10,1.2,630.8048,71.72,1.1676E5,5,24200,Yashin_004_20230531_ZR_DY-3.raw,1,1,Q53HL1|Q53HL1_HUMAN:sp|P19105|ML12A_HUMAN:sp|O14950|ML12B_HUMAN:J3QRS3|J3QRS3_HUMAN,,,PEAKS DB
LQNELDNVSTL-LEEAEKK,48.98,2072.0637,18,1.2,691.6960,73.10,5.8946E4,5,24858,Yashin_004_20230531_ZR_DY-3.raw,1,1,sp|P35580|MYH10_HUMAN:sp|P35580-5|MYH10_HUMAN:A0A8I5KZ38|A0A8I5KZ38_HUMAN:sp|P35580-2|MYH10_HUMAN:sp|P35580-3|MYH10_HUMAN:sp|P35580-4|MYH10_HUMAN,,,PEAKS DB
ELLTRELPSFLGKR,46.48,1657.9514,14,1.5,553.6586,71.80,1.1967E6,5,24225,Yashin_004_20230531_ZR_DY-3.raw,3,3,sp|P26447|S10A4_HUMAN,,,PEAKS DB
SM(+15.99)EAEM(+15.99)IQLQEELAAAER,46.29,2079.9453,18,0.0,694.3224,71.48,7.5847E4,5,24094,Yashin_004_20230531_ZR_DY-3.raw,1,1,A0A024R1N1|A0A024R1N1_HUMAN:sp|P35579|MYH9_HUMAN:A0A8I5KWT8|A0A8I5KWT8_HUMAN:A0A0U4BW16|A0A0U4BW16_HUMAN:sp|P35579-2|MYH9_HUMAN:Q6ZNL4|Q6ZNL4_HUMAN,Oxidation (M),M2:Oxidation (M):1000.00 M6:Oxidation (M):1000.00,PEAKS DB
SGGGGGGGLGSGG-SIRS,45.78,1318.6226,17,1.4,660.3195,37.93,4.7419E5,5,10723,Yashin_004_20230531_ZR_DY-3.raw,1,1,sp|P35527|K1C9_HUMAN,,,PEAKS DB

TTGIVM(+15.99)DSGDGVTHTVPIYEGYAL-
PHAILR,45.49,3198.6018,30,1.7,800.6591,72.46,8.8661E5,5,24551,Yashin_004_20230531_ZR_DY-
3.raw,1,1,A0A2R8Y793|A0A2R8Y793_HUMAN:E7EVS6|E7EVS6_HUMAN:B4DW52|B4DW52_HU-
MAN:I3L4N8|I3L4N8_HUMAN:B4E3A4|B4E3A4_HUMAN:Q8WVW5|Q8WVW5_HU-
MAN:A0A804GS07|A0A804GS07_HUMAN:sp|P60709|ACTB_HUMAN:Q53GK6|Q53GK6_HU-
MAN:Q1KLZ0|Q1KLZ0_HUMAN:Q53G76|Q53G76_HUMAN:Q53G99|Q53G99_HU-
MAN:sp|P63261|ACTG_HUMAN:I3L3I0|I3L3I0_HUMAN:I3L1U9|I3L1U9_HU-
MAN:A0A6Q8PFE4|A0A6Q8PFE4_HUMAN:B3KWQ3|B3KWQ3_HUMAN:Q6PJ43|Q6PJ43_HU-
MAN:B7ZAP6|B7ZAP6_HUMAN:A0A2R8YEA7|A0A2R8YEA7_HUMAN:Q562L9|Q562L9_HU-
MAN:Q562X9|Q562X9_HUMAN:Q562N8|Q562N8_HUMAN:Q562P0|Q562P0_HU-
MAN:Q562Z7|Q562Z7_HUMAN:Q562L5|Q562L5_HUMAN:Q562R8|Q562R8_HU-
MAN:Q562N0|Q562N0_HUMAN:Q562Y6|Q562Y6_HUMAN:Q562U2|Q562U2_HU-
MAN:Q562Z6|Q562Z6_HUMAN:Q562S0|Q562S0_HUMAN:A0AUL6|A0AUL6_HU-
MAN:Q562M5|Q562M5_HUMAN:Q562N2|Q562N2_HUMAN:Q562Y8|Q562Y8_HU-
MAN:Q562M3|Q562M3_HUMAN,Oxidation (M),M6:Oxidation (M):1000.00,PEAKS DB
GETGPAGPAG-
PVGPVGAR,44.89,1545.7899,18,1.7,773.9036,52.26,1.0942E6,5,16413,Yashin_004_20230531_ZR_DY-
3.raw,1,1,sp|P02452|CO1A1_HUMAN:D3DTX7|D3DTX7_HUMAN,,,PEAKS DB
LQQELDDLTVDLD-
HQR,44.33,1936.9490,16,2.1,646.6583,71.15,2.0483E5,5,23924,Yashin_004_20230531_ZR_DY-
3.raw,1,1,sp|P35580|MYH10_HUMAN:sp|P35580-5|MYH10_HUMAN:A0A8I5KZ38|A0A8I5KZ38_HU-
MAN:sp|P35580-2|MYH10_HUMAN:sp|P35580-3|MYH10_HUMAN:sp|P35580-4|MYH10_HU-
MAN,,,PEAKS DB
IRELESQISELQEDLE-
SER,43.92,2302.1287,19,2.1,768.3851,72.81,0,5,24717,Yashin_004_20230531_ZR_DY-
3.raw,0,0,A0A024R1N1|A0A024R1N1_HUMAN:sp|P35579|MYH9_HU-
MAN:A0A8I5KWT8|A0A8I5KWT8_HUMAN:A0A0U4BW16|A0A0U4BW16_HU-
MAN:Q86XU5|Q86XU5_HUMAN:Q99529|Q99529_HUMAN,,,PEAKS DB
LDPHLVLDQLR,43.81,1317.7405,11,2.9,659.8794,72.24,1.7217E5,5,24433,Yashin_004_20230531_ZR_
DY-3.raw,2,2,A0A024R1N1|A0A024R1N1_HUMAN:sp|P35579|MYH9_HU-
MAN:A0A8I5KWT8|A0A8I5KWT8_HUMAN:A0A0U4BW16|A0A0U4BW16_HU-
MAN:Q86XU5|Q86XU5_HUMAN:sp|P35579-2|MYH9_HUMAN:sp|P35580|MYH10_HU-
MAN:sp|P35580-5|MYH10_HUMAN:A0A8I5KZ38|A0A8I5KZ38_HUMAN:sp|P35580-2|MYH10_HU-
MAN:sp|P35580-3|MYH10_HUMAN:sp|P35580-4|MYH10_HUMAN:B4E3S1|B4E3S1_HUMAN,,,PEAKS
DB
TDEAAFQK,43.57,908.4240,8,2.3,909.4333,33.33,5.1511E8,5,8943,Yashin_004_20230531_ZR_DY-
3.raw,5,5,sp|P26447|S10A4_HUMAN:Q5Q9Z3|Q5Q9Z3_HUMAN,,,PEAKS DB
TFHKYSGKEGDKFK,42.29,1670.8416,14,1.4,418.7183,30.26,2.6036E6,5,7941,Yashin_004_20230531_Z
R_DY-3.raw,2,2,sp|P26447|S10A4_HUMAN,,,PEAKS DB
GYSFT-
TTAER,41.75,1131.5197,10,1.5,566.7679,52.78,1.7192E5,5,16631,Yashin_004_20230531_ZR_DY-
3.raw,1,1,A0A2R8Y793|A0A2R8Y793_HUMAN:E7EVS6|E7EVS6_HUMAN:B4DW52|B4DW52_HU-
MAN:I3L4N8|I3L4N8_HUMAN:B4E3A4|B4E3A4_HUMAN:Q8WVW5|Q8WVW5_HU-
MAN:A0A804GS07|A0A804GS07_HUMAN:sp|P60709|ACTB_HUMAN:Q53GK6|Q53GK6_HU-
MAN:Q1KLZ0|Q1KLZ0_HUMAN:Q53G76|Q53G76_HUMAN:Q53G99|Q53G99_HU-
MAN:sp|P63261|ACTG_HUMAN:I3L3I0|I3L3I0_HUMAN:I3L1U9|I3L1U9_HU-

MAN:A0A6Q8PFE4|A0A6Q8PFE4_HUMAN:B4E335|B4E335_HUMAN:B4DVQ0|B4DVQ0_HU-
MAN:B3KWQ3|B3KWQ3_HUMAN:Q6PJ43|Q6PJ43_HUMAN:B7ZAP6|B7ZAP6_HU-
MAN:A0A2R8YEA7|A0A2R8YEA7_HUMAN:V9HVZ7|V9HVZ7_HUMAN,,,PEAKS DB
YSGKEGDKFK,41.41,1157.5717,10,1.3,579.7939,25.19,8.9437E5,5,6323,Yashin_004_20230531_ZR_DY
-3.raw,1,1,sp|P26447|S10A4_HUMAN,,,PEAKS DB
GVVGPQGAR,40.46,839.4613,9,1.9,420.7387,30.66,5.68E5,5,8056,Yashin_004_20230531_ZR_DY-
3.raw,1,1,sp|P08123|CO1A2_HUMAN:A0A0S2Z3H5|A0A0S2Z3H5_HU-
MAN:A0A384MDU2|A0A384MDU2_HUMAN:A0A0S2Z3K0|A0A0S2Z3K0_HU-
MAN:B4DN66|B4DN66_HUMAN,,,PEAKS DB
GEAGAAGPAGPAGPR,39.92,1234.6053,15,1.0,618.3105,33.83,3.0007E5,5,9133,Yashin_004_2023053
1_ZR_DY-3.raw,1,1,sp|P08123|CO1A2_HUMAN:A0A0S2Z3H5|A0A0S2Z3H5_HU-
MAN:A0A384MDU2|A0A384MDU2_HUMAN,,,PEAKS DB
RTDEAAFQK,39.54,1064.5250,9,4.1,533.2720,29.53,4.6034E8,5,7668,Yashin_004_20230531_ZR_DY-
3.raw,2,2,sp|P26447|S10A4_HUMAN:Q5Q9Z3|Q5Q9Z3_HUMAN,,,PEAKS DB
AG-
FAGDDAPR,39.51,975.4410,10,1.5,488.7285,39.77,2.9787E5,5,11457,Yashin_004_20230531_ZR_DY-
3.raw,1,1,A0A2R8Y793|A0A2R8Y793_HUMAN:E7EVS6|E7EVS6_HUMAN:B4DW52|B4DW52_HU-
MAN:I3L4N8|I3L4N8_HUMAN:B4E3A4|B4E3A4_HUMAN:Q8WVW5|Q8WVW5_HU-
MAN:A0A804GS07|A0A804GS07_HUMAN:sp|P60709|ACTB_HUMAN:Q53GK6|Q53GK6_HU-
MAN:Q1KLZ0|Q1KLZ0_HUMAN:Q53G76|Q53G76_HUMAN:Q53G99|Q53G99_HU-
MAN:sp|P63261|ACTG_HUMAN:I3L3I0|I3L3I0_HUMAN:I3L1U9|I3L1U9_HU-
MAN:B4E335|B4E335_HUMAN:B4DVQ0|B4DVQ0_HUMAN:G5E9R0|G5E9R0_HU-
MAN:K7EM38|K7EM38_HUMAN:I3L3R2|I3L3R2_HUMAN:J3KT65|J3KT65_HU-
MAN:Q13707|Q13707_HUMAN:B3KUD3|B3KUD3_HUMAN:B7Z6P1|B7Z6P1_HU-
MAN:A6NL76|A6NL76_HUMAN:B3KW67|B3KW67_HUMAN:A8K3K1|A8K3K1_HU-
MAN:F8WCH0|F8WCH0_HUMAN:C9JTX5|C9JTX5_HUMAN:C9JUM1|C9JUM1_HU-
MAN:C9JZR7|C9JZR7_HUMAN:F8WB63|F8WB63_HUMAN:B8ZZJ2|B8ZZJ2_HU-
MAN:C9JFL5|C9JFL5_HUMAN:F6UVQ4|F6UVQ4_HUMAN:F6QUT6|F6QUT6_HU-
MAN:B7Z6I1|B7Z6I1_HUMAN:Q7Z7J6|Q7Z7J6_HUMAN:L0R5C4|L0R5C4_HUMAN,,,PEAKS DB
VAPEEHPVLL-
TEAPLNPK,39.20,1953.0570,18,0.7,652.0267,69.29,9.2812E5,5,23070,Yashin_004_20230531_ZR_DY-
3.raw,1,1,A0A2R8Y793|A0A2R8Y793_HUMAN:E7EVS6|E7EVS6_HUMAN:B4DW52|B4DW52_HU-
MAN:I3L4N8|I3L4N8_HUMAN:B4E3A4|B4E3A4_HUMAN:Q8WVW5|Q8WVW5_HU-
MAN:A0A804GS07|A0A804GS07_HUMAN:sp|P60709|ACTB_HUMAN:Q53GK6|Q53GK6_HU-
MAN:Q1KLZ0|Q1KLZ0_HUMAN:Q53G76|Q53G76_HUMAN:Q53G99|Q53G99_HU-
MAN:sp|P63261|ACTG_HUMAN:I3L3I0|I3L3I0_HUMAN:I3L1U9|I3L1U9_HU-
MAN:A0A6Q8PFE4|A0A6Q8PFE4_HUMAN:B4E335|B4E335_HUMAN:B4DVQ0|B4DVQ0_HU-
MAN:Q562L9|Q562L9_HUMAN:Q562X9|Q562X9_HUMAN:Q562N8|Q562N8_HU-
MAN:Q562P0|Q562P0_HUMAN:Q562Z7|Q562Z7_HUMAN:Q562L5|Q562L5_HU-
MAN:Q562R8|Q562R8_HUMAN:Q562N0|Q562N0_HUMAN:Q562Y6|Q562Y6_HU-
MAN:Q562U2|Q562U2_HUMAN:Q562Z6|Q562Z6_HUMAN:Q562S0|Q562S0_HU-
MAN:G5E9R0|G5E9R0_HUMAN:K7EM38|K7EM38_HUMAN:I3L3R2|I3L3R2_HU-
MAN:J3KT65|J3KT65_HUMAN:Q562P9|Q562P9_HUMAN:Q562N4|Q562N4_HU-
MAN:Q562Z4|Q562Z4_HUMAN:Q562L6|Q562L6_HUMAN:Q562N6|Q562N6_HU-
MAN:Q562V5|Q562V5_HUMAN,,,PEAKS DB
LTRELPSFLGKR,39.14,1415.8248,12,1.8,472.9497,64.99,2.9684E6,5,21360,Yashin_004_20230531_ZR_
DY-3.raw,1,1,sp|P26447|S10A4_HUMAN,,,PEAKS DB

HKYSGKEGDKFK,38.71,1422.7255,12,2.7,356.6896,20.48,4.4802E6,5,5016,Yashin_004_20230531_ZR_
DY-3.raw,2,2,sp|P26447|S10A4_HUMAN,,,PEAKS DB
KYSGKEGDKFK,38.33,1285.6666,11,1.9,429.5636,21.95,4.4622E5,5,5404,Yashin_004_20230531_ZR_D
Y-3.raw,1,1,sp|P26447|S10A4_HUMAN,,,PEAKS DB
HGLSEG-
WKETEK,38.25,1399.6731,12,1.5,467.5657,40.35,3.601E5,5,11704,Yashin_004_20230531_ZR_DY-
3.raw,2,2,sp|Q6UWP8|SBSN_HUMAN,,,PEAKS DB
KDLYANTVLSGGTTM(+15.99)YPGIADR,37.73,2358.1526,22,0.9,787.0588,69.64,4.0993E5,5,23242,Yash
in_004_20230531_ZR_DY-
3.raw,1,1,A0A2R8Y793|A0A2R8Y793_HUMAN:E7EVS6|E7EVS6_HUMAN:B4DW52|B4DW52_HUMAN:
I3L4N8|I3L4N8_HUMAN:B4E3A4|B4E3A4_HUMAN:Q8WVW5|Q8WVW5_HUMAN:A0A804GS07|A0A
804GS07_HUMAN:sp|P60709|ACTB_HUMAN:Q53GK6|Q53GK6_HUMAN:Q1KLZ0|Q1KLZ0_HUMAN:
Q53G76|Q53G76_HUMAN:Q53G99|Q53G99_HUMAN:sp|P63261|ACTG_HUMAN:A0A6Q8PFE4|A0A
6Q8PFE4_HUMAN:B4E335|B4E335_HUMAN:B4DVQ0|B4DVQ0_HUMAN:B3KWQ3|B3KWQ3_HUMAN
:Q6PJ43|Q6PJ43_HUMAN:B7ZAP6|B7ZAP6_HUMAN:V9HVZ7|V9HVZ7_HUMAN:A4UCT3|A4UCT3_HU
MAN:A5GZ75|A5GZ75_HUMAN:Q96DE1|Q96DE1_HUMAN:Q96FU6|Q96FU6_HUMAN,Oxidation
(M),M15:Oxidation (M):1000.00,PEAKS DB
TRELPSFLGKR,37.46,1302.7407,11,1.6,435.2549,59.39,1.4522E7,5,19171,Yashin_004_20230531_ZR_
DY-3.raw,3,3,sp|P26447|S10A4_HUMAN,,,PEAKS DB
AQYEDIAQK,36.70,1064.5138,9,3.2,533.2659,40.31,3.9545E5,5,11709,Yashin_004_20230531_ZR_DY-
3.raw,1,1,H6VRF8|H6VRF8_HUMAN:sp|P04264|K2C1_HUMAN:H6VRG0|H6VRG0_HU-
MAN:H6VRG3|H6VRG3_HUMAN:H6VRG2|H6VRG2_HUMAN:H6VRF9|H6VRF9_HU-
MAN:H6VRG1|H6VRG1_HUMAN,,,PEAKS DB
ESGSGHSSGLGHR,36.45,1266.5701,13,1.4,634.2932,17.92,9.69E4,5,4370,Yashin_004_20230531_ZR_
DY-3.raw,2,2,sp|Q86YZ3|HORN_HUMAN,,,PEAKS DB
TFHKYSGKEGDK,36.13,1395.6782,12,0.5,466.2336,20.56,6.8228E5,5,5046,Yashin_004_20230531_ZR_
DY-3.raw,2,2,sp|P26447|S10A4_HUMAN,,,PEAKS DB
VIQYLAYVASSHK,35.81,1477.7928,13,1.3,493.6055,70.39,4.3357E4,5,23583,Yashin_004_20230531_Z
R_DY-3.raw,1,1,A0A024R1N1|A0A024R1N1_HUMAN:sp|P35579|MYH9_HU-
MAN:A0A8I5KWT8|A0A8I5KWT8_HUMAN:Q86XU5|Q86XU5_HUMAN:sp|P35579-2|MYH9_HU-
MAN:B4E3S1|B4E3S1_HUMAN:A0A8I5KYI1|A0A8I5KYI1_HUMAN:Q5BKV1|Q5BKV1_HU-
MAN:A0A8I5KU92|A0A8I5KU92_HUMAN:Q9UMJ0|Q9UMJ0_HUMAN,,,PEAKS DB
GGGGGGGLGSGG-
SIR,35.75,1144.5585,15,0.5,573.2868,37.78,5.638E5,5,10648,Yashin_004_20230531_ZR_DY-
3.raw,1,1,sp|P35527|K1C9_HUMAN,,,PEAKS DB
GFSGLDGAK,35.41,850.4185,9,1.1,426.2170,48.82,3.0288E5,5,15072,Yashin_004_20230531_ZR_DY-
3.raw,1,1,sp|P02452|CO1A1_HUMAN:Q6LAN8|Q6LAN8_HUMAN:H9C5C5|H9C5C5_HUMAN,,,PEAKS
DB
SGGGGGGGLGSGG-
SIRSS,35.25,1405.6545,18,1.1,703.8353,36.58,2.3487E5,5,10220,Yashin_004_20230531_ZR_DY-
3.raw,1,1,sp|P35527|K1C9_HUMAN,,,PEAKS DB
IAQLEEELEEEQGN-
TELINDR,35.24,2471.1663,21,1.3,824.7305,71.98,1.1099E5,5,24331,Yashin_004_20230531_ZR_DY-
3.raw,1,1,A0A024R1N1|A0A024R1N1_HUMAN:sp|P35579|MYH9_HU-
MAN:A0A8I5KWT8|A0A8I5KWT8_HUMAN:A0A0U4BW16|A0A0U4BW16_HUMAN:sp|P35579-
2|MYH9_HUMAN:Q6ZNL4|Q6ZNL4_HUMAN,,,PEAKS DB
VLDFEHFLPM(+15.99)LQT-
VAK,34.40,1902.9913,16,0.9,635.3383,73.94,7.3294E5,5,25257,Yashin_004_20230531_ZR_DY-

3.raw,1,1,sp|P60660|MYL6_HUMAN:F8W1R7|F8W1R7_HUMAN:sp|P60660-2|MYL6_HU-
MAN:G8JLA2|G8JLA2_HUMAN:J3KND3|J3KND3_HUMAN:G3V1V0|G3V1V0_HU-
MAN:B7Z6Z4|B7Z6Z4_HUMAN:G3V1Y7|G3V1Y7_HUMAN:Q6IBG5|Q6IBG5_HU-
MAN:F8VPF3|F8VPF3_HUMAN:F8VZU9|F8VZU9_HUMAN:F8W180|F8W180_HUMAN,Oxidation
(M),M10:Oxidation (M):1000.00,PEAKS DB
ELPSFLGK,34.24,889.4908,8,2.1,890.5000,70.80,2.329E8,5,23747,Yashin_004_20230531_ZR_DY-
3.raw,3,3,sp|P26447|S10A4_HUMAN,,,PEAKS DB
RTDE-
AAFQ(+.98)K,33.53,1065.5090,9,1.4,533.7625,31.96,1.1978E6,5,8492,Yashin_004_20230531_ZR_DY-
3.raw,1,1,sp|P26447|S10A4_HUMAN:Q5Q9Z3|Q5Q9Z3_HUMAN,Deamidation (NQ),Q8:Deamidation
(NQ):1000.00,PEAKS DB
GKRTDE-
AAFQK,33.45,1249.6415,11,4.9,417.5565,26.04,2.3374E7,5,6564,Yashin_004_20230531_ZR_DY-
3.raw,1,1,sp|P26447|S10A4_HUMAN,,,PEAKS DB
EAFQLFDR,33.17,1024.4978,8,1.5,513.2570,72.12,3.7037E5,5,24374,Yashin_004_20230531_ZR_DY-
3.raw,1,1,sp|P60660|MYL6_HUMAN:F8W1R7|F8W1R7_HUMAN:sp|P60660-2|MYL6_HU-
MAN:G8JLA2|G8JLA2_HUMAN:J3KND3|J3KND3_HUMAN:G3V1V0|G3V1V0_HU-
MAN:B7Z6Z4|B7Z6Z4_HUMAN:G3V1Y7|G3V1Y7_HUMAN,,,PEAKS DB
YSGKEGDKF-
KLN,33.00,1384.6986,12,2.1,693.3580,42.67,2.5858E7,5,12733,Yashin_004_20230531_ZR_DY-
3.raw,4,4,sp|P26447|S10A4_HUMAN,,,PEAKS DB
YSGKEGDKF-
KLN(+.98)K,32.99,1513.7776,13,1.5,757.8972,38.99,2.1699E5,5,11160,Yashin_004_20230531_ZR_DY-
3.raw,1,1,sp|P26447|S10A4_HUMAN,Deamidation (NQ),N12:Deamidation (NQ):1000.00,PEAKS DB
ELKELLTR,32.80,1000.5917,8,0.6,1001.5995,55.39,1.4796E8,5,17634,Yashin_004_20230531_ZR_DY-
3.raw,2,2,sp|P26447|S10A4_HUMAN:Q5Q9Z3|Q5Q9Z3_HUMAN,,,PEAKS DB
ELPSFLGKR,32.23,1045.5920,9,2.7,523.8047,69.40,3.1447E8,5,23092,Yashin_004_20230531_ZR_DY-
3.raw,3,3,sp|P26447|S10A4_HUMAN,,,PEAKS DB
LTRELPSFLGK,31.36,1259.7238,11,3.0,420.9164,69.54,9.1225E6,5,23153,Yashin_004_20230531_ZR_D
Y-3.raw,2,2,sp|P26447|S10A4_HUMAN,,,PEAKS DB
HVLVTLGEK,31.32,994.5811,9,1.1,498.2984,50.89,2.7278E5,5,15918,Yashin_004_20230531_ZR_DY-
3.raw,1,1,sp|P60660|MYL6_HUMAN:F8W1R7|F8W1R7_HUMAN:sp|P60660-2|MYL6_HU-
MAN:G8JLA2|G8JLA2_HUMAN:J3KND3|J3KND3_HUMAN:G3V1V0|G3V1V0_HU-
MAN:B7Z6Z4|B7Z6Z4_HUMAN:G3V1Y7|G3V1Y7_HUMAN:Q6IBG5|Q6IBG5_HU-
MAN:F8VPF3|F8VPF3_HUMAN:F8VZU9|F8VZU9_HUMAN:F8W180|F8W180_HU-
MAN:F8VXL3|F8VXL3_HUMAN:H0YI43|H0YI43_HUMAN,,,PEAKS DB
TDE-
AAFQ(+.98)K,31.25,909.4080,8,1.9,455.7121,36.18,5.4726E5,5,10023,Yashin_004_20230531_ZR_DY-
3.raw,1,1,sp|P26447|S10A4_HUMAN:Q5Q9Z3|Q5Q9Z3_HUMAN,Deamidation (NQ),Q7:Deamidation
(NQ):1000.00,PEAKS DB
STFHKYSGK,30.69,1053.5243,9,1.6,527.7703,23.46,7.5616E4,5,5840,Yashin_004_20230531_ZR_DY-
3.raw,1,1,sp|P26447|S10A4_HUMAN,,,PEAKS DB
GPAGPQGPR,30.61,835.4300,9,2.3,418.7232,25.20,6.2148E5,5,6368,Yashin_004_20230531_ZR_DY-
3.raw,1,1,sp|P02452|CO1A1_HUMAN:D3DTX7|D3DTX7_HUMAN:B7Z4S2|B7Z4S2_HUMAN,,,PEAKS
DB
ALEEALEA-
KEEFER,30.55,1662.8101,14,1.1,555.2779,66.74,6.4547E4,5,22108,Yashin_004_20230531_ZR_DY-

3.raw,1,1,sp|P35580|MYH10_HUMAN:sp|P35580-5|MYH10_HUMAN:A0A8I5KZ38|A0A8I5KZ38_HU-MAN:sp|P35580-2|MYH10_HUMAN:sp|P35580-3|MYH10_HUMAN:sp|P35580-4|MYH10_HU-MAN,,,PEAKS DB
GPSGPQGIR,30.47,867.4562,9,1.4,434.7360,32.92,6.1641E5,5,8804,Yashin_004_20230531_ZR_DY-3.raw,1,1,sp|P08123|CO1A2_HUMAN:A0A0S2Z3H5|A0A0S2Z3H5_HU-MAN:A0A384MDU2|A0A384MDU2_HUMAN,,,PEAKS DB
RTDE-AAFQKP,30.34,1161.5778,10,7.4,581.8005,44.34,4.7461E5,5,13380,Yashin_004_20230531_ZR_DY-3.raw,1,1,Q5Q9Z3|Q5Q9Z3_HUMAN,,,PEAKS DB
TRELPSFLGK,29.53,1146.6396,10,1.2,383.2209,66.29,7.6135E7,5,21878,Yashin_004_20230531_ZR_DY-3.raw,2,2,sp|P26447|S10A4_HUMAN,,,PEAKS DB
YSPDVLK,29.01,820.4330,7,0.6,821.4408,55.73,4.6106E6,5,17801,Yashin_004_20230531_ZR_DY-3.raw,1,1,B3KXF2|B3KXF2_HUMAN:A0A804HJA4|A0A804HJA4_HUMAN:sp|Q5VYK3|ECM29_HU-MAN:J3KN16|J3KN16_HUMAN,,,PEAKS DB
ELPSFLGKRTD,28.60,1261.6666,11,3.7,421.5644,66.49,1.0455E7,5,21933,Yashin_004_20230531_ZR_DY-3.raw,1,1,sp|P26447|S10A4_HUMAN,,,PEAKS DB
FHKYSGK,28.22,865.4446,7,1.6,433.7303,19.08,6.4826E5,5,4652,Yashin_004_20230531_ZR_DY-3.raw,1,1,sp|P26447|S10A4_HUMAN,,,PEAKS DB
YSGKEGDKF,27.93,1029.4767,9,4.4,515.7479,32.31,2.0242E7,5,8592,Yashin_004_20230531_ZR_DY-3.raw,1,1,sp|P26447|S10A4_HUMAN,,,PEAKS DB
FHKYSGKEGDKFK,27.92,1569.7939,13,2.3,524.2731,29.09,7.2158E5,5,7529,Yashin_004_20230531_ZR_DY-3.raw,1,1,sp|P26447|S10A4_HUMAN,,,PEAKS DB
ELLTRELPSFL,27.90,1316.7340,11,2.2,659.3757,74.33,5.1814E4,5,25462,Yashin_004_20230531_ZR_DY-3.raw,1,1,sp|P26447|S10A4_HUMAN,,,PEAKS DB
SSSSGSVGESSSKGP,27.78,1338.5898,15,3.6,670.3046,24.80,2.5579E6,5,6188,Yashin_004_20230531_ZR_DY-3.raw,1,1,sp|P13645|K1C10_HUMAN:A0A1B0GVI3|A0A1B0GVI3_HUMAN,,,PEAKS DB
TFHKYSGK,27.69,966.4923,8,0.7,484.2538,20.66,7.5282E5,5,5061,Yashin_004_20230531_ZR_DY-3.raw,1,1,sp|P26447|S10A4_HUMAN,,,PEAKS DB
SGKEGDKF-KLN(+.98)K,27.67,1350.7142,12,1.6,676.3655,31.86,6.5971E5,5,8461,Yashin_004_20230531_ZR_DY-3.raw,1,1,sp|P26447|S10A4_HUMAN,Deamidation (NQ),N11:Deamidation (NQ):1000.00,PEAKS DB
SGFSSVSVSR,26.70,1011.4985,10,0.5,506.7567,49.21,0,5,15222,Yashin_004_20230531_ZR_DY-3.raw,0,0,B4DRR0|B4DRR0_HUMAN:B4DRU6|B4DRU6_HUMAN:A8K2I0|A8K2I0_HU-MAN:A0A0S2Z428|A0A0S2Z428_HUMAN,,,PEAKS DB
ELPSFLGKRTDE,26.11,1390.7092,12,1.8,696.3632,66.61,1.4155E7,5,22049,Yashin_004_20230531_ZR_DY-3.raw,1,1,sp|P26447|S10A4_HUMAN,,,PEAKS DB
VVSLSSAK,26.05,789.4596,8,0.8,790.4675,35.31,9.5702E4,5,9731,Yashin_004_20230531_ZR_DY-3.raw,1,1,A0A0A0MSY1|A0A0A0MSY1_HUMAN:sp|B1ANS9-2|WDR64_HU-MAN:sp|B1ANS9|WDR64_HUMAN:A0A0C4DG52|A0A0C4DG52_HUMAN:H0Y6L4|H0Y6L4_HU-MAN,,,PEAKS DB
RTDE-AAFQ(+.98)KLMSN(+.98)L,25.80,1624.7766,14,9.9,542.6049,62.99,1.2111E5,5,20635,Yashin_004_20230531_ZR_DY-3.raw,1,1,sp|P26447|S10A4_HUMAN,Deamidation (NQ),Q8:Deamidation (NQ):1000.00 N13:Deamidation (NQ):1000.00,PEAKS DB
